# Traitement orthopédique d'une fracture pathologique du fémur sur malformation veineuse: à propos d'un cas

**DOI:** 10.11604/pamj.2015.22.3.7765

**Published:** 2015-09-03

**Authors:** Soufiane Guelzim, Omar Lamrani, Mohammed Kharmaz, Abdo Lahlou, Mohammed Elouadghiri, Ahmed El Bardouni, Mustapha Mahfoud, Mohammed Saleh Berrada, Mouradh El Yaccoubi

**Affiliations:** 1 Service de Traumatologie Orthopédie, CHU Ibn Sina, Rabat, Maroc

**Keywords:** Fracture pathologique, phléboscanner, fracture diaphysaire, pathological fracture, CT venography,, diaphyseal fracture

## Abstract

Les malformations vasculaires artérioveineuse, veineuse ou lymphatique représentent des défects localisés dans la morphogénèse vasculaire. Elles peuvent survenir dans tous les organes, mais prédominent au niveau de membres, plus souvent dans la peau, les espaces celluleux ou les muscles. Le bilan de nombreuses malformations a été transformé par l'utilisation de l'angioscanner avec reconstruction ou de l'IRM. Les auteurs rapportent un cas de fracture pathologique du fémur proximal gauche sur malformation veineuse, chez une patiente de 35 ans. Le diagnostic a été porté sur un faisceau d'arguments cliniques et paracliniques. La radiographie standard a montré une fracture diaphysaire du tiers supérieur du fémur gauche sur os pathologique. L'IRM de la cuisse gauche ainsi qu'un phléboscanner des membres inférieurs ont objectivé un aspect en faveur d'une malformation veineuse. Vu le déplacement minime de la fracture et le risque très important de saignement peropératoire, la patiente a bénéficié d'un traitement orthopédique; l’évolution a été simple, marquée par une consolidation au sixième mois.

## Introduction

Les malformations veineuses (MV) sont des dysembryogénies du système vasculaire veineux. Elles envahissent n'importe quel tissu ou type d'organe. Cliniquement, une MV cutanée se caractérise par une masse bleutée compressible à la palpation. Des phlébolithes sont fréquemment présents. Sa symptomatologie est fonction de sa localisation et de sa taille. Le plus souvent sporadique et isolée, la MV peut être associée à d'autres malformations et faire partie d'un syndrome; le plus connu étant le syndrome de Klippel-Trenaunay ou malformation capillarolymphaticoveineuse associée à une hypertrophie du membre atteint. Le diagnostic de MV est souvent évoqué suite à la coloration bleutée de la lésion. Néanmoins, d'autres anomalies tumorales ou malformatives peuvent présenter ce même symptôme. Les plus fréquents sont le naevus bleu, la malformation lymphatique hémorragique, l'hémangiome sous-cutané, la dilatation veineuse superficielle ou la présence d'un réseau veineux collatéral anormal stigmate d'une sténose sous-jacente [1]. L'angiomatose veineuse osseuse ostéolytique est rare, souvent systématisée (sur un membre par exemple) ou diffuse, elle peut être isolée, purement osseuse, associée à un angiome veineux superficiel, ou encore à un hémolymphangiome en regard [2]. Les auteurs rapportent un cas de fracture pathologique sur angiodysplasie veineuse de la cuisse gauche.

## Patient et observation

**Figure 1:**
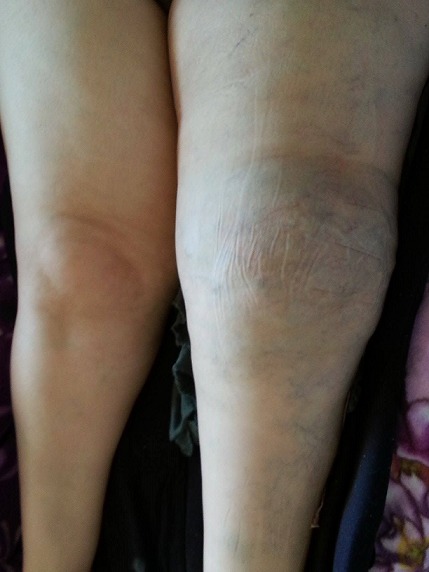
Image clinique du membre inférieur gauche montrant une hypertrophie musculaire de la cuisse gauche avec de nombreuses varicosités diffuses

**Figure 2:**
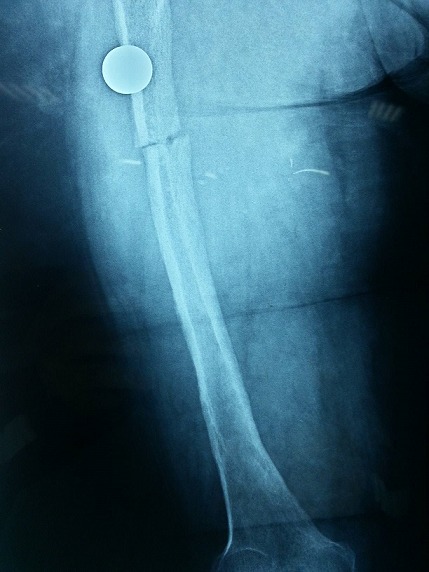
Radiographie standard de la cuisse gauche montrant un fémur dysplasique déformé et aminci siège d'une fracture pathologique à son tiers supérieur peu déplaçée

**Figure 3:**
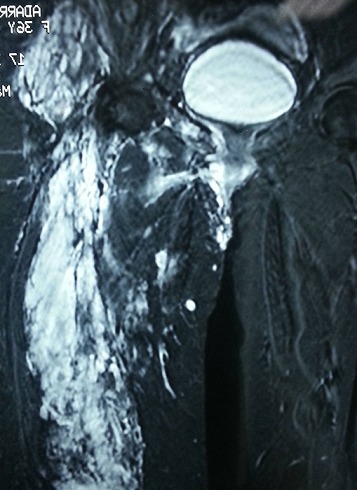
Image IRM de l'angiodysplasie veineuse superficielle et profonde des parties molles de la cuisse

**Figure 4:**
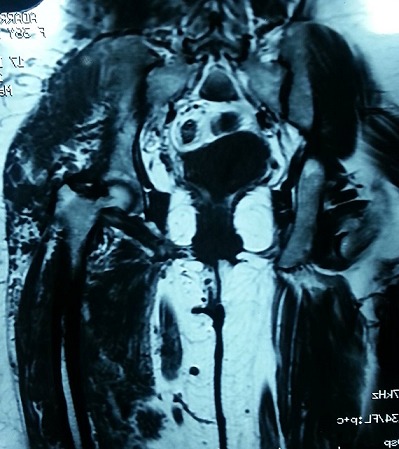
Image IRM de l'angiodysplasie veineuse étendue jusqu'aux muscles fessiers

Il s'agit d'une patiente âgée de 35 ans, présentant une malformation veineuse de la cuisse gauche depuis son enfance pour laquelle elle n'a jamais consulté, jusqu’à son hospitalisation au service de traumato-orthopédie pour une fracture du fémur gauche suite à un traumatisme minime. L'examen clinique objectivait une hypertrophie avec grande circulation veineuse collatérale intéressant tout le membre inférieur gauche avec douleur et impotence fonctionnelle (Figure 1). La radiographie standard a révélé un fémur gauche dysplasique, déformé et aminci, siège d'une fracture diaphysaire au niveau de son tiers supérieur, très peu déplaçée (Figure 2). Une IRM ainsi qu'un phléboscanner ont été réalisés pour préciser la nature de cette malformation vasculaire et son étendue et qui ont objectivé une angiodysplasie veineuse superficielle et profonde intéressant toutes les parties molles de la cuisse gauche très étendue jusqu'aux muscles fessiers (Figure 3, Figure 4). Vu le risque important de saignement peropératoire et le déplacement minime de la fracture du fémur siégeant sur un os dysplasique déformé et aminci, la patiente a bénéficié d'un traitement orthopédique associé à un traitement médical par antalgiques et anticoagulants (HBPM); l’évolution a été simple, marquée par une consolidation au sixième mois.

## Discussion

Les malformations vasculaires représentent des défects localisés dans la morphogenèse vasculaire. Elles peuvent survenir dans tous les organes, mais prédominent au niveau de membres, plus souvent dans la peau, les espaces celluleux ou les muscles [3]. Les membres inférieurs en sont le site privilégié. Elles sont parfois visibles à la naissance et se développent en fonction de la croissance corporelle et ne se manifestent que tardivement à l'adolescence. De multiples perturbations squelettiques ont été rapportées chez des enfants porteurs de malformations vasculaires, liées aux perturbations hémodynamiques de la sphère musculaire et ostéopériostée et à l'hypervascularisation de la région des cartilages fertiles. Ces altérations peuvent affecter la forme, la taille ou la densité osseuse. Elles peuvent ainsi aboutir à une distorsion, une hypertrophie ou, plus rarement, une hypotrophie en longueur ou en diamètre du squelette; Mais la révélation de cette lésion par une fracture pathologique est exceptionnelle [4]. Cliniquement, une MV cutanée se caractérise par une masse bleutée compressible à la palpation. Des phlébolithes sont fréquemment présents. Sa symptomatologie est fonction de sa localisation et de sa taille. Le plus souvent sporadique et isolée, la MV peut être associée à d'autres malformations et faire partie d'un syndrome; le plus connu étant le syndrome de Klippel-Trenaunay ou malformation capillarolymphaticoveineuse associée à une hypertrophie du membre atteint. Le diagnostic de MV est souvent évoqué suite à la coloration bleutée de la lésion. Néanmoins, d'autres anomalies tumorales ou malformatives peuvent présenter ce même symptôme. Les plus fréquents sont le naevus bleu, la malformation lymphatique hémorragique, l'hémangiome sous-cutané, la dilatation veineuse superficielle ou la présence d'un réseau veineux collatéral anormal stigmate d'une sténose sous-jacente [1]. La radiographie standard évalue le retentissement osseux. Elle peut contribuer au diagnostic lorsqu'elle trouve des phlébolithes, qui sont inconstants, mais pathognomoniques de la stase et de la thrombose dans ces poches veineuses. Parfois, elle peut montrer un épaississement périosté, ou des ostéolyses osseuses associées [5].

L’écho-Doppler permet un bilan précis des anomalies des troncs veineux profonds et des anomalies tissulaires; une hypervascularisation est cependant trouvée dans les topographies où la malformation prédomine. Le scanner avec ou sans injection, peut parfois constituer un complément pré-thérapeutique. Hormis le fait qu'il visualise les phlébolithes, il montre plus précisément les limites de la malformation veineuse (MV) et les rapports anatomiques de voisinage. L'IRM est le seul examen performant, permettant sur les coupes axiales en spin-écho de T2, le diagnostic et la localisation topographique précise. Son intérêt est d'identifier les localisations profondes des MV surtout dans leur topographie intra-musculaire ou intra-articulaire (genou). Elle a supplanté le scanner avec injection car plus performante sur le plan diagnostique et pré-thérapeutique (2). L'artériographie reste utile pour faire le bilan des complications tronculaires des MV ou lorsqu'une chirurgie est programmée, afin de mieux identifier les pédicules nourriciers et les voies de drainage. Elle est, bien sûr, le préalable à de rares embolisations. Le traitement non chirurgical est soit un complément du traitement chirurgical, soit une alternative possible à ce dernier: contention élastique, drainage pneumatique intermittent, embolisation et/ou sclérothérapie. Les MV diffuses relèvent rarement d'une prise en charge chirurgicale, en raison de l'association aux éléments malformatifs superficiels, d'une destruction tissulaire sous-jacente, l'absence d'un plan de clivage rend l'exérèse délabrante [6]. L’évolution se fait toujours vers une vraie ou une fausse récidive dans la mesure où la malformation évolue lentement tout au long de la vie [2]. La chirurgie est indiquée pour les MV localisées, une exérèse musculaire partielle est possible.Les indications de la chirurgie orthopédique sont limitées aux cas de complications. Elles concernent aussi la prévention d'un allongement anormal du membre atteint, la prévention ou la correction des attitudes vicieuses. Le chirurgien orthopédiste a le plus souvent recours à des épiphysiodèses sur le membre touché par la malformation veineuse, à des allongements sur le membre sain ou à des ténotomies sur les rétractions fibreuses musculaires en cas de flessum de genou ou un varus équin de la cheville afin de corriger ces déformations [2].

## Conclusion

La fracture pathologique sur malformation veineuse reste exceptionnelle. Le diagnostic est souvent évoqué cliniquement devant la coloration bleutée de la lésion. La radiographie standard évalue le retentissement osseux et l'IRM précise la lésion, sa topographie et son étendue. Le traitement est pluridisciplinaire faisant intervenir la chirurgie vasculaire, la radiologie interventionnelle et la chirurgie orthopédique qui intervient surtout pour améliorer la symptomatologie fonctionnelle (corriger une désaxation, une déformation ou une rétraction) et pour traiter les complications (fracture pathologique…). Il faut souligner en cas de chirurgie orthopédique le risque important de saignement peropératoire d'où le choix du traitement orthopédique concernant notre patiente.
